# Safety and Efficacy of Semaglutide in Patients With Chronic Kidney Disease, With or Without Type 2 Diabetes: A Systematic Review and Meta‐Analysis

**DOI:** 10.1002/edm2.70136

**Published:** 2025-11-23

**Authors:** Ali Abdullah, F. N. U. Sagreeka, Gurdas Alias Aniket, Rohan Lal, F. N. U. Geeta, Anusha Bai, Ghazi Uddin Ahmed, Ahmed Asad Raza, Varisha Fatima Shaikh, Owais Sanaullah, Syeda Elezeh Sabahat, Ahzam Khan Ghori, Seema Habib Bhutto, Mahir Tesfaye

**Affiliations:** ^1^ Jinnah Sindh Medical University Karachi Pakistan; ^2^ Ghulam Muhammad Mahar Medical College Sukkur Pakistan; ^3^ Chandka Medical College Larkana Pakistan; ^4^ Dow Medical College Karachi Pakistan; ^5^ Jinnah Medical and Dental College Karachi Pakistan; ^6^ Addis Ababa University Addis Ababa Ethiopia

**Keywords:** cardiovascular outcomes, chronic kidney disease, GLP‐1 receptor agonist, major adverse cardiovascular events, major kidney adverse events, meta‐analysis, renal outcomes, semaglutide, type 2 diabetes mellitus

## Abstract

**Background:**

Chronic kidney disease (CKD) affects over half a billion people globally and significantly increases the risk of cardiovascular complications, particularly in those with type 2 diabetes mellitus (T2DM). Although semaglutide, a glucagon‐like peptide‐1 receptor agonist, has shown favourable cardiorenal effects in T2DM patients, prior meta‐analyses were limited by small sample sizes and few studies. This updated meta‐analysis includes both diabetic and non‐diabetic CKD patients, incorporates recently published RCTs and addresses gaps in the literature to enhance result generalizability.

**Methods:**

MEDLINE, Embase and Cochrane CENTRAL were searched from inception to May 2025 following PRISMA and AMSTAR guidelines. Studies comparing semaglutide with placebo or standard care in adults (≥ 18 years) with CKD, with or without T2DM were included. Primary outcomes included cardiovascular mortality, major adverse cardiovascular events (MACE), major kidney‐related adverse events, nonfatal myocardial infarction and nonfatal stroke. Risk of bias was assessed using Cochrane RoB 2.0.

**Results:**

Five RCTs involving 12,785 participants were included. The findings showed that semaglutide substantially decreased major kidney‐related adverse events (defined as a composite outcome encompassing the onset of kidney failure (including long‐term dialysis, kidney transplantation or a sustained eGFR reduction to < 15 mL/min/1.73 m^2^), a sustained 50% or greater reduction in eGFR from baseline, or death due to kidney‐related causes) (RR: 0.79; 95% CI: 0.71–0.87; *p* < 0.00001; *I* ^2^ = 0%), cardiovascular mortality (RR: 0.74; 95% CI: 0.62–0.88; *p* = 0.0008; *I* ^2^ = 36%) and MACE (defined as a composite of cardiovascular death, nonfatal myocardial infarction and nonfatal stroke) (RR: 0.78; 95% CI: 0.70–0.87; *p* < 0.00001; *I* ^2^ = 0%).

**Conclusion:**

Semaglutide demonstrates a favourable safety profile and significant cardiorenal benefits in CKD patients, with or without T2DM. Further research is needed to confirm its effects in non‐diabetic CKD populations.

**Trial Registration:**

International Prospective Register of Systematic Reviews (PROSPERO) registration number: CRD420251019235

## Graphical abstract

Semaglutide offers dual kidney and heart protection in patients with chronic kidney disease.

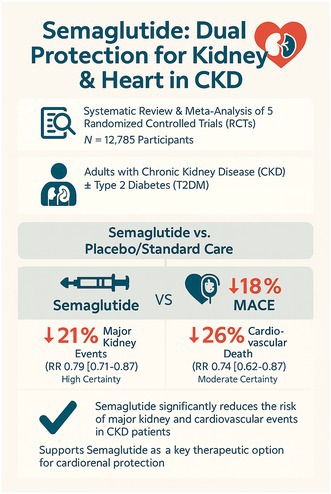

## 1 Introduction

With a global prevalence of around 9% and affecting over half a billion people, chronic kidney disease (CKD) is the 12th leading cause of death worldwide. Individuals with CKD face a significantly greater risk for cardiovascular complications, progression to kidney failure and mortality [1, 2]. Around 40% of the population aged above 70 years is affected by early‐stage CKD (stages 1–3), accounting for the majority of the disease's public health burden [3, 4]. The leading cause of CKD is type 2 diabetes mellitus (T2DM), affecting nearly 50% of the patients [5].

In recent years, treatment strategies for CKD have primarily focused on controlling associated risk factors, including hyperglycemia, dyslipidemia and blood pressure, ideally managed with medications modulating the renin‐angiotensin‐aldosterone system (RAAS). However, the emergence of cardiovascular outcome trials (CVOT) has revealed that glucagon‐like peptide‐1 receptor agonists (GLP‐1RA) and sodium‐glucose cotransporter 2 inhibitors (SGLT‐2i) offer renoprotective benefits in addition to their glucose‐lowering effects [6, 7].

Compared to other drugs in its class, semaglutide, a GLP‐1RA, has demonstrated remarkable effectiveness in managing blood glucose levels, promoting weight loss and decreasing cardiovascular and metabolic risks. It has been shown to significantly improve glycemic parameters, reduce mortality and hospital admissions related to heart failure, lower the incidence of cardiovascular adverse effects, and slow the progression of renal function decline [8, 9].

A recent systematic review and meta‐analysis synthesised data from 3 randomised controlled trials (RCTs) involving 10,013 patients and evaluated the safety and efficacy of semaglutide in patients with T2DM and CKD [10]. The study found that semaglutide significantly reduced the risk of both major cardiovascular adverse events (MACE) and kidney‐related complications compared to placebo, maintaining a favourable safety profile in T2DM patients with CKD. However, despite these benefits, the same meta‐analysis exhibited a number of limitations, such as a lack of data on CKD patients without diabetes and moderate heterogeneity in outcomes. The generalizability of findings was also limited owing to the small sample size (3 RCTs) and short follow‐up durations.

Given these limitations, an updated meta‐analysis incorporating studies including both diabetic and non‐diabetic patients with CKD was necessary for a more comprehensive assessment across a broader patient population. In addition to the three RCTs included in the previous meta‐analysis, we incorporated two additional trials in our study: one focusing on the role of semaglutide in CKD patients without diabetes and the other assessing its effects on cardiovascular outcomes.

## 2 Methods

Our systematic review and meta‐analysis adhered to the Preferred Reporting Items for Systematic Reviews and Meta‐Analyses (PRISMA) guidelines. Additionally, the study also followed the AMSTAR (Assessing the Methodological Quality of Systematic Reviews) guidelines. Relevant studies were identified that evaluated the safety and efficacy of semaglutide in individuals with CKD, with or without T2DM, through a comprehensive search.

### 2.1 Data Sources and Search Strategy

A systematic search was conducted across electronic databases, which included MEDLINE, Embase and Cochrane CENTRAL, from their inception until May 2024. A combination of Medical Subject Headings (MeSH) terms and keywords was employed by the search strategy. The keywords included ‘semaglutide,’ ‘type 2 diabetes mellitus,’ ‘chronic kidney disease,’ ‘randomized controlled trial,’ and ‘placebo.’ Boolean operators (AND, OR) were used to refine search outcomes, and database‐specific filters, which included limiting to RCTs in humans and those conducted in the English language, were applied to ensure methodological rigour. Further studies were identified by reviewing reference lists of pertinent systematic reviews and meta‐analyses. Furthermore, clinical trial registries as well as conference abstracts were examined to identify unpublished or ongoing trials. Table 1 contains the detailed search strategy.

**TABLE 1:** Baseline characteristics.

Study	Total sample	Body weight—kg		Body mass index		Glycated haemoglobin level—%		Systolic blood pressure—mm Hg		Diastolic blood pressure—mm Hg		Myocardial infarction—no. (%)		Heart failure—no. (%)		Estimated GFR—mL/min/1.73 m2		Percentage with T2DM	Diuretics		SGLT2 inhibitor	
		Semaglutide	Placebo	Semaglutide	Placebo	Semaglutide	Placebo	Semaglutide	Placebo	Semaglutide	Placebo	Semaglutide	Placebo	Semaglutide	Placebo	Semaglutide	Placebo		Semaglutide	Placebo	Semaglutide	Placebo
Perkovic et al. (2024) [11]	3533	89.5 ± 19.8	89.8 ± 21.2	31.9 ± 6.1	32.0 ± 6.5	7.8 ± 1.3	7.8 ± 1.3	138.9 ± 16.1	138.4 ± 15.4	76.8 ± 10.0	76.1 ± 10.0	405 (22.9)	403 (22.8)	342 (19.4)	336 (19.0)	46.9 ± 15.6	47.1 ± 14.7	100%	870	910	277	273
Marso et al. (2016) [12]	3297	92.35 ± 20.7	91.85 ± 20.55	—	—	8.7 ± 1.45	8.7 ± 1.5	135.95 ± 17.5	135.3 ± 16.85	77 ± 10.0	77.1 ± 10.05	530 (32.15)	542 (32.85)	381 (23.1)	396 (24.05)	—	—	100%	—	—	—	—
Husain et al. (2019) [13]	3183	91.0 ± 21.4	90.8 ± 21.0	32.3 ± 6.6	32.3 ± 6.4	8.2 ± 1.6	8.2 ± 1.6	135 ± 18	136 ± 18	76 ± 10	76 ± 10	—	—	—	—	74 ± 21	74 ± 21	100%	—	—	—	—
Pratley et al. (2024) [14]	3532	90.45 (78.2–104.55)	90.45 (78.2–104.55)	32.05 (28.4–36.35)	32.05 (28.4–36.35)	7.9 (7.1–8.8)	7.5 (6.8–8.5)	138.0 (129.0–147.0)	138.0 (128.0–148.0)	78.0 (71.0–84.0)	77.0 (70.0–83.0)	266 (15.1)	522 (29.6)	506 (28.7)	825 (46.7)	45.0 (36.0–56.0)	45.0 (36.0–56.0)	100%	519	591	82	85
Apperloo et al. (2025) [15]	101	108.7 (20)	101.2 (15)	37.0 (7.0)	35.4 (3.5)	5.6 (0.3)	5.7 (0.3)	—	—	—	—	—	—	—	—	65.8 (26)	64.3 (25)	0%	12	17	11	8

### 2.2 Inclusion and Exclusion Criteria

#### 2.2.1 Study Selection and Data Extraction

The inclusion criteria consisted of RCTs that involved adult participants (≥ 18 years) diagnosed with both T2DM and CKD. The studies that were eligible compared semaglutide against either a placebo or standard care and reported at least one predetermined clinical outcome. Primary outcomes included major kidney‐related adverse events, MACE, cardiovascular mortality, nonfatal myocardial infarction and nonfatal stroke. Major kidney‐related adverse events were defined as a composite outcome encompassing the onset of kidney failure (including long‐term dialysis, kidney transplantation, or a sustained eGFR reduction to < 15 mL/min/1.73 m^2^), a sustained 50% or greater reduction in eGFR from baseline, or death due to kidney‐related causes. MACE was defined as a composite of cardiovascular death, nonfatal myocardial infarction and nonfatal stroke, in accordance with standard definitions used across the included trials. All‐cause mortality, serious adverse events, hospitalisation due to unstable angina or heart failure, and the use of cardiovascular medications, were the secondary outcomes. Observational studies, case reports, case series, reviews and editorials were excluded. Studies were also excluded if they lacked a control group. Conference abstracts were excluded from this review to ensure inclusion of only peer‐reviewed studies. Lastly, studies that failed to report relevant clinical outcomes were also excluded. Two reviewers assessed the titles and abstracts of all retrieved studies to determine eligibility, independently. Full‐text articles were obtained for studies that appeared to meet the inclusion criteria or when inclusion remained uncertain. Any disagreements between reviewers were resolved through discussion or consultation with a third reviewer. Extracted data included study characteristics, patient demographics, interventions, outcomes and risk‐of‐bias assessments using a standardised data extraction form.

### 2.3 Risk of Bias Assessment

The quality of the included RCTs was assessed using the Cochrane Risk‐of‐Bias methodology for Randomised Trials (RoB 2.0) [16]. This evaluates possible bias in five areas: outcome measurement, missing outcome data, variations from intended treatments, the randomization procedure and the selection of reported outcomes. Bias was categorised as ‘low risk,’ ‘some concerns,’ or ‘high risk’ for each domain. Any disagreements were settled by the reviewers' consensus.

### 2.4 Statistical Analysis

The statistical analyses were performed using Review Manager (Revman) software version 5.4 (The Cochrane Collaboration, Copenhagen, Denmark). To account for any heterogeneity among studies, risk ratios (RRs) with 95% CIs were computed using a random‐effects model for dichotomous outcomes. The chi‐squared test was used to measure heterogeneity, and the *I* ^2^ statistic was used to quantify it. Low, moderate and high heterogeneity were indicated by *I* ^2^ values of 25%, 50% and 75%, respectively [17]. A significant *p*‐value was defined as less than 0.05. Because there were < 10 papers, the publication bias was not evaluated using funnel plots. A number of studies have used this approach [18, 19, 20].

## 3 Results

**FIGURE 1:**
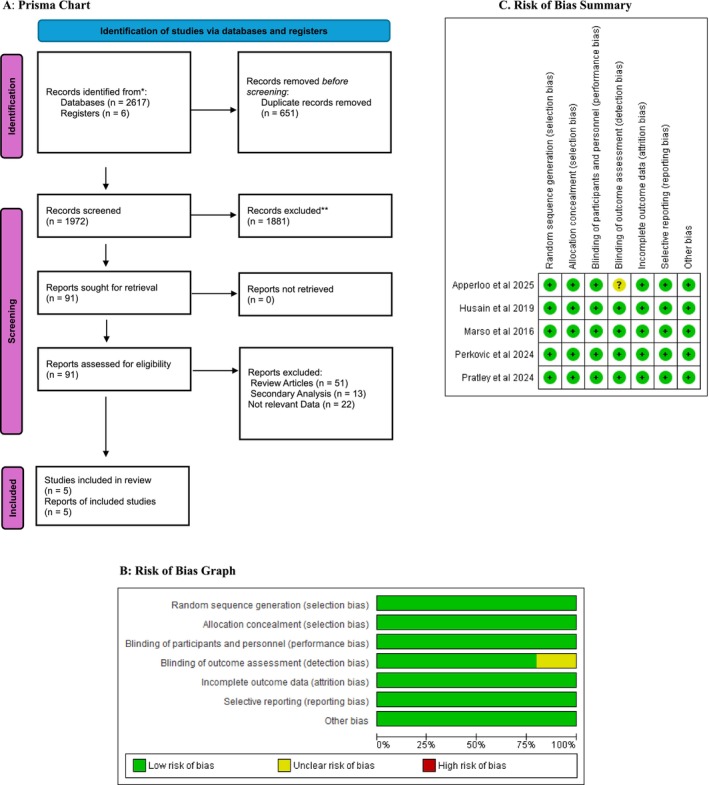
(A) Prisma chart. (B) Risk of bias graph. (C) Risk of bias summary.

A total of five RCTs comprising 12,785 patients comorbid with T2DM and CKD were included according to the inclusion and exclusion criteria. The PRISMA flowchart depicts the study selection process. From 2617 records identified (2611 from databases and 6 from registers) in total, 651 duplicates were removed, and narrowed down 1972 titles/abstracts were screened, with 1881 excluded. After assessing 91 full‐text articles, five studies met all eligibility criteria and were included in the study (Figure 1A).

### 3.1 Demographics and Baselines

The included studies assessed a predominantly older population (mean age 64–67 years), except for Apperloo et al. [15], which had a younger cohort (54.9 ± 12 years in the semaglutide group). Gender distribution showed a higher proportion of male participants across studies. Baseline bodyweight ranged from 89.5 kg to 108.7 kg, with semaglutide groups generally having slightly higher body weights. BMI values varied from 31.9 to 37.0 kg/m^2^, with the highest observed in Apperloo et al. [15] (37.0 ± 7.0 in the semaglutide group). Baseline glycated haemoglobin (HbA1c) levels were comparable between treatment groups across studies, ranging from 5.6% to 8.7%. Mean systolic blood pressure was consistently around 135–138 mmHg, while diastolic blood pressure ranged from 76 to 78 mmHg, with no notable differences between treatment groups. All five included studies clearly specified the diabetes status of their enrolled populations. Four trials—Perkovic et al. [11], Marso et al. [12], Husain et al. [13] and Pratley et al. [14]—exclusively enrolled patients with T2DM, resulting in a 100% prevalence of T2DM in those study populations [11, 12, 13, 14]. In contrast, Apperloo et al. [15] was the only trial to include patients with CKD without diabetes, enrolling a cohort composed entirely of non‐diabetic participants (0% T2DM) [15].

### 3.2 Risk of Bias Assessment

Cochrane's Risk‐of‐Bias Tool was implemented to calculate bias risk across shortlisted studies. Overall, a low risk of bias was demonstrated in key domains, including random sequence generation, allocation concealment, blinding of participants and personnel, and selective reporting. Nevertheless, specific trials raised concerns on blinding of outcome assessment, which could be a potential source of bias (Figure 1B,C and Table S2). Furthermore, the certainty of evidence for primary outcomes was evaluated using the GRADE approach, and a summary table has been included (Table S3).

### 3.3 Primary Outcomes

#### 3.3.1 Cardiovascular Mortality

**FIGURE 2:**
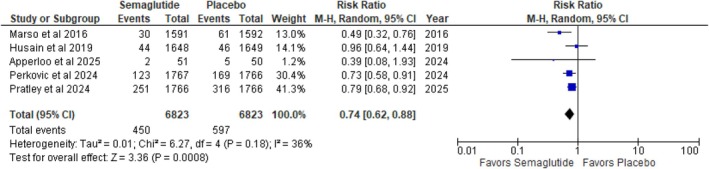
Death from cardiovascular causes (cardiovascular mortality).

Subjects receiving semaglutide showed a statistically significant reduction in cardiovascular mortality relative to placebo (RR: 0.74; 95% CI: 0.62 to 0.88; *p* = 0.0008; *I* ^2^ = 36%) (Figure 2). Relative risk reduction in death from cardiovascular causes was calculated to be 26%. However, the moderate heterogeneity (*I* ^2^ = 36%) indicates some variability among studies.

#### 3.3.2 Major Adverse Cardiovascular Events (MACE)

**FIGURE 3:**
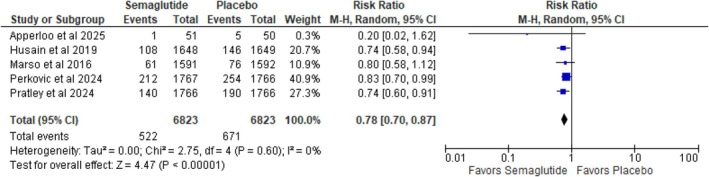
Major adverse cardiovascular events.

The incidence of MACE also drastically decreased on receiving semaglutide (RR: 0.78; 95% CI: 0.70 to 0.87; *p* < 0.00001; *I* ^2^ = 0%), indicating a 22% relative risk reduction (Figure 3). The absence of heterogeneity (*I* ^2^ = 0%) indicates consistency among the included studies, strengthening the reliability of this finding.

#### 3.3.3 Major Kidney Adverse Events

**FIGURE 4:**
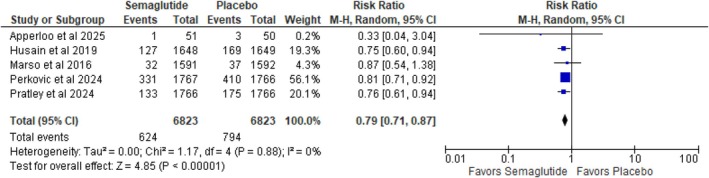
Major kidney adverse events.

A notable fall in major kidney adverse events was noted upon analysis of patients treated with semaglutide in contrast to those on placebo (RR: 0.79; 95% CI: 0.71 to 0.87; *p* < 0.00001; *I* ^2^ = 0%) (Figure 4). Relative risk reduction score stands at 20%. The absence of heterogeneity (*I* ^2^ = 0%) indicates consistency among the included studies, strengthening the reliability of this finding.

#### 3.3.4 Nonfatal Myocardial Infarction

**FIGURE 5:**
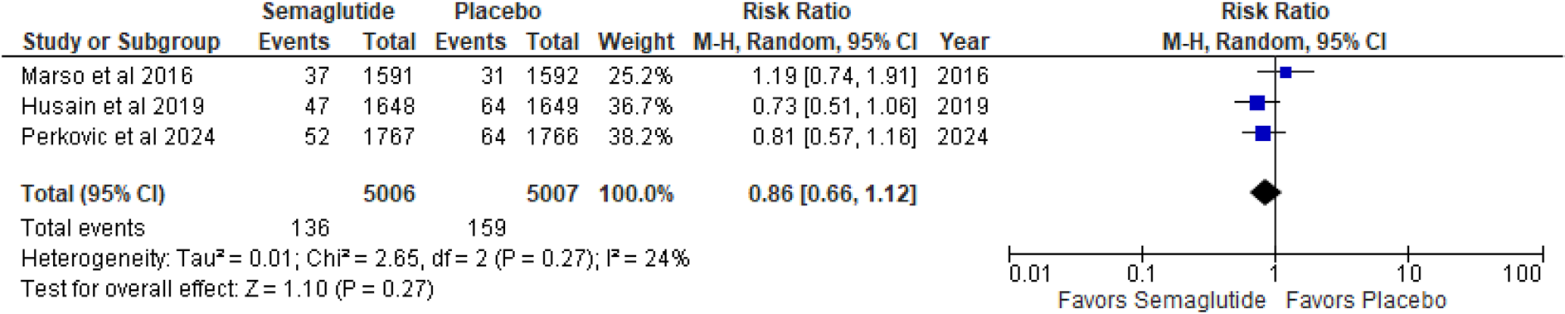
Nonfatal myocardial infarction.

Semaglutide group was less likely to develop nonfatal myocardial infarction when compared to placebo, but this difference did not reach statistical significance (RR: 0.86; 95% CI: 0.66 to 1.12; *p* = 0.27; *I* ^2^ = 24%) (Figure 5). Relative risk reduction was 14%, but the wide confidence interval crossing unity indicates uncertainty in the effect estimate. The low heterogeneity among studies (*I* ^2^ = 24%) suggests consistent findings.

#### 3.3.5 Nonfatal Stroke

**FIGURE 6:**
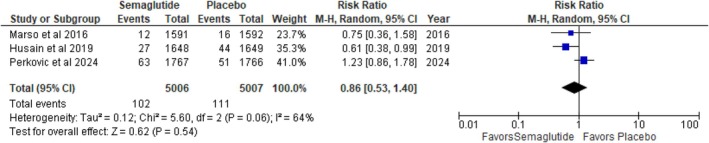
Nonfatal stroke.

No statistically significant difference in nonfatal stroke was observed between the semaglutide and placebo groups (RR: 0.86; 95% CI: 0.53–1.40; *p* = 0.54; *I* ^2^ = 64%) (Figure 6). The high heterogeneity (*I* ^2^ = 64%) indicates substantial variability among studies; however, the non‐significant heterogeneity test (*p* = 0.06) suggests that this variability may not be impactful. A sensitivity analysis was performed by sequentially removing individual studies. We found that the study by Perkovic et al. influenced the outcome variability. After excluding this study, the results favored semaglutide (RR: 0.65; 95% CI: 0.44–0.97; *p* = 0.04; *I* ^2^ = 0%) (Figure S1).

### 3.4 Secondary Outcomes

#### 3.4.1 All‐Cause Mortality

A non‐significant trend was observed in all‐cause mortality in the semaglutide group (RR: 0.80; 95% CI: 0.68 to 0.93; *p* = 0.004; *I* ^2^ = 44%) (Figure S2). This indicates a 20% relative risk reduction, with moderate heterogeneity (*I* ^2^ = 44%), which is not statistically significant (*p* = 0.15), indicating relatively consistent findings across studies.

#### 3.4.2 Serious Adverse Events

A sloping trend of serious adverse events was observed in semaglutide subjects when compared to placebo (RR: 0.86; 95% CI: 0.74 to 0.99; *p* = 0.04; *I* ^2^ = 87%) (Figure S3A). A favourable safety profile for semaglutide was highlighted by a 14% relative risk reduction. The high heterogeneity indicates substantial variability among studies, which may impact the reliability of the overall estimate. A sensitivity analysis was performed by sequentially removing individual studies. We found that the study by Pratley et al. influenced the outcome variability. After excluding this study, the results became more significant towards semaglutide (RR: 0.92; 95% CI: 0.87–0.98; *p* = 0.01; *I* ^2^ = 14%) (Figure S3B).

#### 3.4.3 Hospitalisation for Unstable Angina

There was no notable difference between the two groups: semaglutide and placebo regarding hospitalizations due to unstable angina (RR: 1.01; 95% CI: 0.55 to 1.84; *p* = 0.98; *I* ^2^ = 27%) (Figure S4). The risk ratio close to 1 suggests no effect, and the wide confidence interval represents limited precision due to minimal event rates. The low heterogeneity (*I* ^2^ = 27%) indicates uniform results across studies.

#### 3.4.4 Hospitalisation for Heart Failure

The rate of hospitalizations for cardiac failure was not affected by semaglutide administration (RR: 1.03; 95% CI: 0.76 to 1.40; *p* = 0.86; *I* ^2^ = 0%) (Figure S5). The lack of heterogeneity (*I* ^2^ = 0%) suggests that the findings are consistent among the included studies. The risk ratio indicates no significant difference between groups.

#### 3.4.5 Use of Cardiovascular Medications

The need for cardiovascular medications was markedly decreased in the semaglutide group (Figure S6). Improved cardiovascular outcomes have potentially led to a reduced need for cardiovascular pharmacological interventions as depicted by a 14% relative risk reduction. The low heterogeneity supports the reliability of this finding.

## 4 Discussion

By synthesising data from five carefully selected RCTs, our meta‐analysis offers a robust, quantitative assessment of the current evidence of semaglutide's safety and efficacy for treating individuals that have chronic kidney disease, with or without diabetes mellitus. Our findings suggest a significant reduction in cardiovascular mortality, major adverse cardiovascular events, and major kidney adverse events among patients that were treated with semaglutide as compared to placebo, while also highlighting its favourable safety profile. The drug's potential to provide comprehensive cardio‐renal protection in this high‐risk population, is elucidated by this as well.

The observed 2% risk reduction in cardiovascular mortality among patients on semaglutide, is particularly compelling, given the elevated cardiovascular risk that is intrinsic to individuals with CKD [1, 2]. The moderate heterogeneity (*I* ^2^ = 36%) suggests some variability in study populations or methodologies, and warrants further investigation into the subgroups that benefit the most. The 22% risk reduction in MACE, further substantiates this, with an absence of heterogeneity (*I* ^2^ = 0%), confirming its consistency across the included studies. Both of these collectively reinforce semaglutide's potential to mitigate the burden of cardiovascular disease in CKD, which aligns with the latest meta‐analysis on this topic, conducted by Ashraf et al., which discovered a 29% decrease in cardiovascular mortality, and a 20% decrease seen in MACE [10]. The risk of nonfatal myocardial infarction and nonfatal stroke incidence did not achieve significant reduction, despite a downward trend, which is also similar to the study by Ashraf et al. [10]. This indicates that additional large‐scale trials with longer follow‐up durations, to assess the impact on specific cardiovascular endpoints, should be carried out. Other studies have also found improved cardiovascular outcomes as a result of GLP‐1RAs [21, 22]. Accordingly, the current ESC guidelines recommend GLP‐1RAs as the first‐line antidiabetic therapy in patients with high cardiovascular risk [23].

The 20% risk reduction in major kidney adverse events exemplifies the favourable impact of semaglutide on renal outcomes. The absence of heterogeneity (*I* ^2^ = 0%) strengthens the confidence in this finding. This observation builds upon the broader understanding that GLP‐1RAs can have renoprotective effects that are beyond standard‐of‐care treatments like RAAS inhibitors and SGLT‐2 inhibitors [24, 25]. Potential pathways explaining this may involve inhibition of progression to microalbuminuria [26], decrease in oxidative stress and inflammation [27, 28], as well as modulation of glomerular pressure. However, the precise mechanisms driving these effects are still under investigation [24]. Some subgroup analyses have recommended a combination therapy of SGLT‐2 inhibitors with GLP‐1RAs; however, no large‐scale trial has been conducted directly to test this [25]. Reduction in major kidney adverse events was also, similarly, found in the study by Ashraf et al. and other studies [10, 11].

A significant reduction of 20% was also observed in all‐cause mortality. Additional trials with longer follow‐ups should be conducted to confirm this due to the moderate heterogeneity (*I* ^2^ = 44%). This has been found previously as well for GLP‐1RAs [29, 30]. Additionally, the fact that hospitalisation rates for unstable angina and heart failure were not significantly different between the groups, suggests that semaglutide's primary benefits may be in long‐term cardiovascular event prevention and not in acute hospitalisation reduction. The drug's favourable safety profile and the potential for reducing the overall burden of cardiovascular disease was supported by the 14% relative reduction in serious adverse events compared to placebo, coupled with the reduced need for cardiovascular medications in the semaglutide group. Substantial heterogeneity (*I* ^2^ = 87%), however, raises concerns regarding variations in adverse event reporting across studies. Previous studies have also indicated a favourable safety profile [30, 31, 32].

Our results are generally in line with the findings of the recent meta‐analysis by Ashraf et al. [10] of three RCTs, which included 10,013 patients. It found a 20% decrease in kidney‐related adverse events (RR: 0.80; 95% CI: 0.71–0.89), a 29% decrease in cardiovascular mortality (RR: 0.71; 95% CI: 0.52–0.97) and a 20% decrease in MACE (RR: 0.80; 95% CI: 0.71–0.91). The main distinctions between the two meta‐analyses are in the number and range of studies included. Our analysis included two extra RCTs, including the complete results of the FLOW trial, which have more recently become available [11, 15]. These discrepancies are the probable explanation for the reduced effect size in our cardiovascular outcomes, that is, a 26% decrease in cardiovascular mortality and an 18% decrease in MACE, compared to Ashraf et al.'s higher estimates. These differences notwithstanding, both of the meta‐analyses exhibit uniform and substantial advantages of semaglutide on cardiovascular and renal outcomes for patients with T2DM and CKD, emphasising its worth in clinical utility for this high‐risk group.

Our findings advocate for semaglutide's role as a valuable therapeutic option for patients with CKD due to its demonstrated cardiovascular and renal benefits. The observed reduction in cardiovascular medication usage also means that semaglutide may decrease the need for intensive pharmacological interventions. This might improve patient quality of life, adherence and healthcare costs [33, 34]. Its favourable safety profile across multiple trials, also advocates for it being a well‐tolerated option for CKD management. Although significant decreases in cardiovascular and kidney outcomes were shown, it must be noted that these results mostly represent results among CKD patients with T2DM. Of the five trials included, four (Perkovic et al. [11], Marso et al. [12], Husain et al. [13] and Pratley et al. [14]) included only diabetic subjects, and only one (Apperloo et al. [15]) included non‐diabetic CKD patients. The total population studied was thus predominantly diabetic which means that the generalizability of these results to non‐diabetic CKD subjects is not certain. We have laid out the number of patients with and without T2DM in each trial in Table 1, and highlight the imperative for randomised trials in the future to formally assess semaglutide in non‐diabetic CKD populations. It should also be noted that unlike some other GLP‐1As, semaglutide has no association with cancer [35]. Semaglutide is also a useful adjunctive treatment for patients with hypertension and obesity [32].

Several limitations in this study must be acknowledged. First, even though five RCTs were included, the sample size still remains limited. The follow‐up durations were also relatively short, which might lead to underestimation of long‐term benefits and risks. Second, patients with CKD with and without diabetes were included, which introduces heterogeneity in baseline characteristics and hence, may influence outcomes. Since the study population was predominantly diabetic, the limited representation of non‐diabetic patients restricts the generalizability of our conclusions to that subgroup. Third, potential bias in the blinding of assessments of outcomes in some studies may also have impacted the findings. Fourth, variations in the definition and reporting of adverse effects across studies introduce uncertainty regarding the overall safety profile. Finally, most of the trials that have been included, were not designed specifically to evaluate renal outcomes, which raises the need for dedicated nephrology‐based trials, as previously stated. Future research should focus on long‐term, large‐scale trials that assess semaglutide's effects across different stages of CKD and comorbidities.

## 5 Conclusion

Semaglutide shows efficacy in reducing cardiovascular and kidney‐related adverse events in CKD patients, with or without diabetes. It also maintains a favourable safety profile and reduces the need for cardiovascular medications. These findings are collectively supportive of its use as a therapeutic strategy for CKD management, with or without diabetes. Long‐term studies, however are needed to confirm its role in reducing rates of hospitalisation and mortality in broader populations of patients.

## Author Contributions

**Ali Abdullah:** conceptualization, project development, data collection, manuscript writing. **F. N. U. Sagreeka:** project development, data collection, manuscript writing. **Gurdas Alias Aniket:** project development, data collection, manuscript writing. **Rohan Lal:** project development, data collection, manuscript writing. **F. N. U. Geeta:** table, manuscript writing. **Anusha Bai:** data analysis, manuscript writing. **Ghazi Uddin Ahmed:** data analysis, manuscript writing. **Ahmed Asad Raza:** figures, data analysis, manuscript writing. **Varisha Fatima Shaikh:** figure, manuscript writing. **Owais Sanaullah:** manuscript writing, and editing. **Syeda Elezeh Sabahat:** supervision, manuscript writing, and editing. **Ahzam Khan Ghori:** figures, manuscript writing. **Seema Habib Bhutto:** manuscript writing, and editing. **Mahir Tesfaye:** supervision, manuscript writing, and editing.

## Conflicts of Interest

The authors declare no conflicts of interest.

## Supporting information


**Table S1:** Search strategy.
**Table S2:** Risk of Bias table.
**Table S3:** GRADE table for primary outcomes.
**Figure S1:** Leave one out analysis for Nonfatal Stroke.
**Figure S2:** Death from any cause (All‐Cause Mortality).
**Figure S3:** (A) Serious Adverse Events. (B) Leave one out analysis for Serious Adverse Events.
**Figure S4:** Unstable Angina Resulting in Hospitalisation.
**Figure S5:** Heart failure resulting in hospitalisation.
**Figure S6:** Cardiovascular medication.

## Data Availability

The data that supports the findings of this study are available in the Supporting Information of this article.
