# Supplementary material for: Safety and Efficacy of Semaglutide in Patients With Chronic Kidney Disease, With or Without Type 2 Diabetes: A Systematic Review and Meta‐Analysis
Source: Endocrinol Diabetes Metab. 2025 Nov 23;8(6):e70136. doi: 10.1002/edm2.70136 (PMC12640882; doi:10.1002/edm2.70136)

**Supplementary Table 1:** Search Strategy

| PubMed/MEDLINE  (290 results) | ("semaglutide"[Supplementary Concept] OR "semaglutide"[All Fields]) AND "T2DM"[All Fields] |
| --- | --- |
| Cochrane Library  (97 results) | Semaglutide AND T2DM |
| Google Scholar  (1,981 results) | Semaglutide AND T2DM |
| Science Direct  (249 results) | Semaglutide AND T2DM |
| Clinicaltrials.gov  (6 results) | Semaglutide AND T2DM |

**Supplementary Table 2: Risk of Bias Table.**

|  | Cochrane Risk-of-Bias Tool | | |
| --- | --- | --- | --- |
|  | Bias | Risk of bias | Author judgement |
| Apperloo et al 2025 | Random sequence generation (selection bias) | Low Risk | Participants were randomized (1:1) to receive semaglutide 2.4 mg per week or placebo. |
|  | Allocation concealment (selection bias) | Low Risk | In this double-blind trial, neither patients nor researchers were aware of the names of individual patients or the assignments they were getting as therapy, despite the lack of a clear description of the allocation concealment process. As a result, the allocation concealment strategy was probably used successfully, reducing the likelihood of biased intervention allocation. |
|  | Blinding of participants and personnel (performance bias) | Low Risk | The study was double-blind, thus, blinding of participants and personnel was implemented. |
|  | Blinding of outcome assessment (detection bias) | Unclear Risk | It is unclear whether outcome assessors were blinded, which could introduce bias in measuring results. |
|  | Incomplete outcome data (attrition bias) | Low Risk | The study had a low dropout rate, reducing the risk of bias from missing data. |
|  | Selective reporting (reporting bias) | Low Risk | There is no evidence of selective outcome reporting, reducing the risk of reporting bias. |
|  | Other bias | Low Risk | No other biases were identified in the study that could significantly impact the results. |
| Husain et al 2019 | Random sequence generation (selection bias) | Low Risk | Patients were randomly assigned (in a 1:1 ratio) to receive a once-daily oral semaglutide (target dose, 14 mg) or placebo. |
|  | Allocation concealment (selection bias) | Low Risk | The study effectively implemented an allocation concealment procedure, ensuring patients and investigators were unaware of specific patient identities or treatment assignments, thereby reducing the risk of biased intervention allocation. |
|  | Blinding of participants and personnel (performance bias) | Low Risk | The study was double-blind, thus, blinding of participants and personnel was implemented. |
|  | Blinding of outcome assessment (detection bias) | Low Risk | The outcome assessment was undertaken by an independent external event-adjudication committee whose members were unaware of the trial-group assignments. |
|  | Incomplete outcome data (attrition bias) | Low Risk | Incomplete outcome data were handled appropriately, minimizing the risk of attrition bias. |
|  | Selective reporting (reporting bias) | Low Risk | There is no evidence of selective outcome reporting, reducing the risk of reporting bias. |
|  | Other bias | Low Risk | No other biases were identified in the study that could significantly impact the results. |
| Marso et al 2016 | Random sequence generation (selection bias) | Low Risk | Patients were randomized in a 1:1:1:1 ratio to receive either 0.5 mg or 1.0 mg of once-weekly subcutaneous semaglutide or volume-matched placebo. |
|  | Allocation concealment (selection bias) | Low Risk | Despite the absence of a clear explanation of the allocation concealment procedure, neither patients nor investigators in this double-blind experiment knew the identities of specific patients or the assignments they were receiving as treatment. Therefore, it is likely that the allocation concealment approach was implemented effectively, lowering the possibility of intervention allocation that is biased. |
|  | Blinding of participants and personnel (performance bias) | Low Risk | The study was double-blind, thus, blinding of participants and personnel was implemented. |
|  | Blinding of outcome assessment (detection bias) | Low Risk | Each outcome was adjudicated in a blinded fashion by an external, independent event-adjudication committee. |
|  | Incomplete outcome data (attrition bias) | Low Risk | Incomplete outcome data were handled appropriately, minimizing the risk of attrition bias. |
|  | Selective reporting (reporting bias) | Low Risk | There is no evidence of selective outcome reporting, reducing the risk of reporting bias. |
|  | Other bias | Low Risk | No other biases were identified in the study that could significantly impact the results. |
| Perkovic et al 2024 | Random sequence generation (selection bias) | Low Risk | Eligible participants were randomly assigned in a 1:1 ratio to receive semaglutide or matching placebo with the use of a central interactive Web based response system. |
|  | Allocation concealment (selection bias) | Low Risk | Patients and investigators were unaware of assignments to study groups. As a result, there are minimal chance of biased intervention allocation because the allocation concealment technique was implemented correctly. |
|  | Blinding of participants and personnel (performance bias) | Low Risk | The study was described as "double-blind," indicating that both participants and personnel were blinded to the treatment allocation. |
|  | Blinding of outcome assessment (detection bias) | Low Risk | Results were adjudicated in a blinded fashion by an event adjudication committee. |
|  | Incomplete outcome data (attrition bias) | Low Risk | Incomplete outcome data were handled appropriately, minimizing the risk of attrition bias because the discontinuation rate of patients is approximately same. |
|  | Selective reporting (reporting bias) | Low Risk | There is no evidence of selective outcome reporting, reducing the risk of reporting bias. |
|  | Other bias | Low Risk | No other biases were identified in the study that could significantly impact the results. |
| Pratley et al 2024 | Random sequence generation (selection bias) | Low Risk | Eligible participants were randomized, in a 1:1 ratio, to receive once-weekly sub cutaneous injections of semaglutide 1.0 mg or matching placebo. |
|  | Blinding of participants and personnel (performance bias) | Low Risk | The study was described as "double-blind," indicating that both participants and personnel were blinded to the treatment allocation. |
|  | Blinding of outcome assessment (detection bias) | Low Risk | The events were adjudicated by an independent adjudication committee in accordance with prespecified criteria. Members of the adjudication committee were blinded to trial group assignment. |
|  | Incomplete outcome data (attrition bias) | Low Risk | Incomplete outcome data were handled appropriately, minimizing the risk of attrition bias because the discontinuation rate of patients is approximately same. |
|  | Selective reporting (reporting bias) | Low Risk | There is no evidence of selective outcome reporting, reducing the risk of reporting bias. |
|  | Other bias | Low Risk | No other biases were identified in the study that could significantly impact the results. |

**Supplementary Table 3:** GRADE Table for Primary Outcomes

| **Outcome** | **No. of Studies (Participants)** | **Relative Effect (RR)** | **95% CI** | **Absolute Effect** | **Certainty of Evidence (GRADE)** | **Comments** |
| --- | --- | --- | --- | --- | --- | --- |
| **Major kidney-related adverse events** | 5 RCTs (12,785) | 0.79 | 0.71 to 0.87 | 47 fewer per 1,000 (from 66 to 25) | ⬤⬤⬤⬤ High | Consistent, precise effect across studies; low heterogeneity (I² = 0%) |
| **Cardiovascular mortality** | 5 RCTs (12,785) | 0.74 | 0.62 to 0.87 | 18 fewer per 1,000 (from 26 to 9) | ⬤⬤⬤◯ Moderate | Moderate heterogeneity (I² = 36%); consistent direction of effect |
| **Major adverse cardiovascular events** | 5 RCTs (12,785) | 0.78 | 0.70 to 0.87 | 28 fewer per 1,000 (from 39 to 15) | ⬤⬤⬤⬤ High | Consistent results with low heterogeneity (I² = 0%) |
| **Nonfatal myocardial infarction** | 3 RCTs (10,013) | 0.86 | 0.66 to 1.12 | 6 fewer per 1,000 (from 17 fewer to 4 more) | ⬤⬤⬤◯ Moderate | Not statistically significant; downgraded for imprecision |
| **Nonfatal stroke** | 3 RCTs (10,013) | 0.86 | 0.53 to 1.40 | 3 fewer per 1,000 (from 15 fewer to 10 more) | ⬤⬤◯◯ Low | High heterogeneity (I² = 64%); downgraded for inconsistency and imprecision |

**Supplementary Figure 1: Leave one out analysis for Nonfatal Stroke**

**
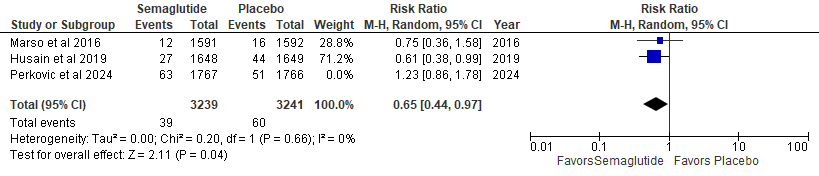
**

**Supplementary Figure 2:** Death from Any Cause (All-Cause Mortality)


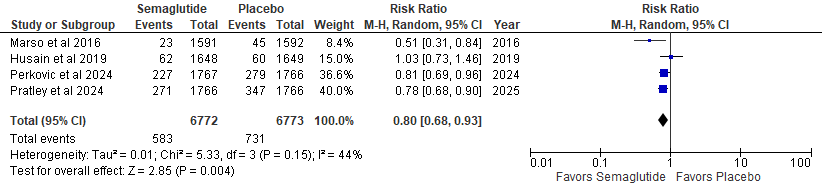


**Supplementary Figure 3B:** Serious Adverse Events

**
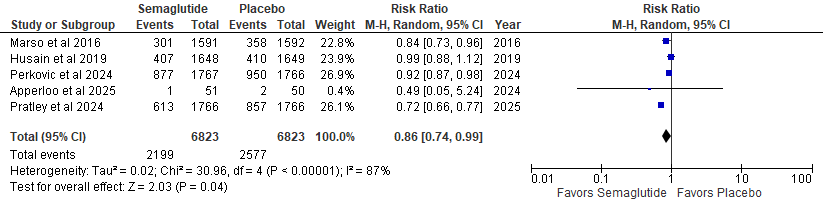
**

**Supplementary Figure 3B: Leave one out analysis for Serious Adverse Events**

**
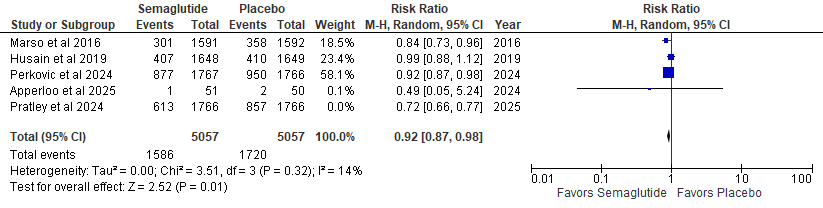
**

**Supplementary Figure 4:** Unstable Angina Resulting in Hospitalization

**
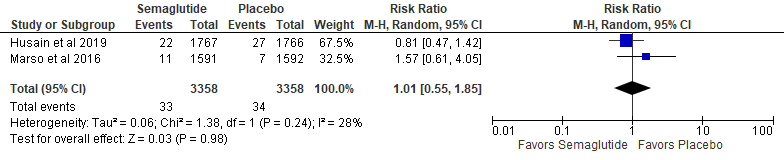
**

**Supplementary Figure 5:** Heart failure Resulting in Hospitalization


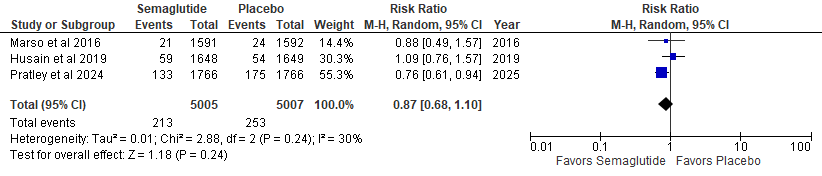


**Supplementary Figure 6:** Cardiovascular Medication


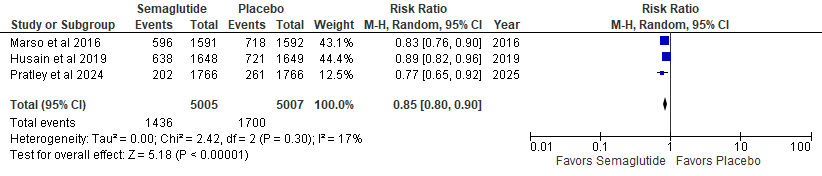

Supplement: Supplementary file 1 — Table S1: Search strategy. Table S2: Risk of Bias table. Table S3: GRADE table for primary outcomes. Figure S1: Leave one out analysis for Nonfatal Stroke. Figure S2: Death from any cause (All‐Cause Mortality). Figure S3: (A) Serious Adverse Events. (B) Leave one out analysis for Serious Adverse Events. Figure S4: Unstable Angina Resulting in Hospitalisation. Figure S5: Heart failure resulting in hospitalisation. Figure S6: Cardiovascular medication. [file EDM2-8-e70136-s001.docx]
